# Research progress of extracellular vesicles in PMOS: molecular mechanisms, diagnostic value, and therapeutic strategies

**DOI:** 10.3389/fendo.2026.1976561

**Published:** 2026-10-20

**Authors:** Lijing Qi, Yue Jiang, Yanhua Han, Cong Feng, Liyan Jia, Yuehui Zhang

**Affiliations:** 1 Gynecology Department, Shenzhen Traditional Chinese Medicine Hospital, Shenzhen, China; 2 Department of Obstetrics and Gynecology, Key Laboratory and Unit of Infertility in Chinese Medicine, First Affiliated Hospital of Heilongjiang University of Traditional Chinese Medicine, Harbin, China

**Keywords:** biomarkers, extracellular vesicles, PCOS, PMOS, polyendocrine metabolic ovarian syndrome, therapeutic targets

## Abstract

Extracellular vesicles (EVs) carry proteins, lipids and nucleic acids that can influence ovarian and systemic metabolic function. This narrative review examines their molecular mechanisms, biomarker potential and experimental therapeutic applications in polyendocrine metabolic ovarian syndrome (PMOS), previously termed polycystic ovary syndrome. Follicular fluid EV cargo varies with disease phenotype and adiposity, but these associations have not established clinically validated diagnostic tests. Follicular fluid collection also requires an invasive procedure and should not be described as a non-invasive screening approach. Experimental studies indicate that selected mesenchymal stromal cell-derived EV preparations can modulate granulosa cell survival, mitochondrial quality control and endocrine function. Adipose stromal cell- and brown adipose tissue-derived preparations additionally affect metabolic pathways, while engineered small EVs can improve delivery of a PI3K agonist in mice. Disease-associated follicular fluid EVs may impair oocyte maturation or promote abnormal granulosa cell proliferation; their effects depend on source, cargo and recipient context. We distinguish PMOS-specific evidence from chemotherapy-related ovarian injury and premature ovarian insufficiency, summarize treatment models, administration and endpoints, and evaluate reporting in selected studies against MISEV2023 recommendations. The evidence remains predominantly preclinical. Incomplete EV characterization, heterogeneous doses and models, limited biodistribution data and uncertain long-term reproductive safety prevent direct extrapolation to patient care.

## 1 Introduction

Polyendocrine metabolic ovarian syndrome (PMOS), previously termed polycystic ovary syndrome (PCOS), encompasses reproductive, endocrine and metabolic abnormalities. The 2026 international naming consensus emphasizes this broader disease spectrum (1). We use PMOS in the narrative while retaining PCOS in original article titles. Phenotypic heterogeneity, including variation in adiposity and androgen status, is particularly relevant when interpreting EV studies. Extracellular vesicles (EVs) are lipid bilayer-delimited particles released by cells. Their size, composition and formation pathways are heterogeneous. In accordance with MISEV2023, “EV” is used as the general term; “small EV” (sEV) is an operational size-based description, commonly referring to particles below approximately 200 nm, rather than proof of endosomal origin. “Exosome” denotes an EV of endosomal origin and should be used only when that origin is established. Size, electron microscopy and tetraspanin expression alone cannot establish exosome biogenesis (2). Original terminology is retained in reference titles, but experimental preparations are described conservatively as EVs or sEVs throughout this review. Endosomal release, plasma membrane budding and apoptotic fragmentation are distinct processes, not an interconversion sequence (**Figure 1**).

**Figure 1:**
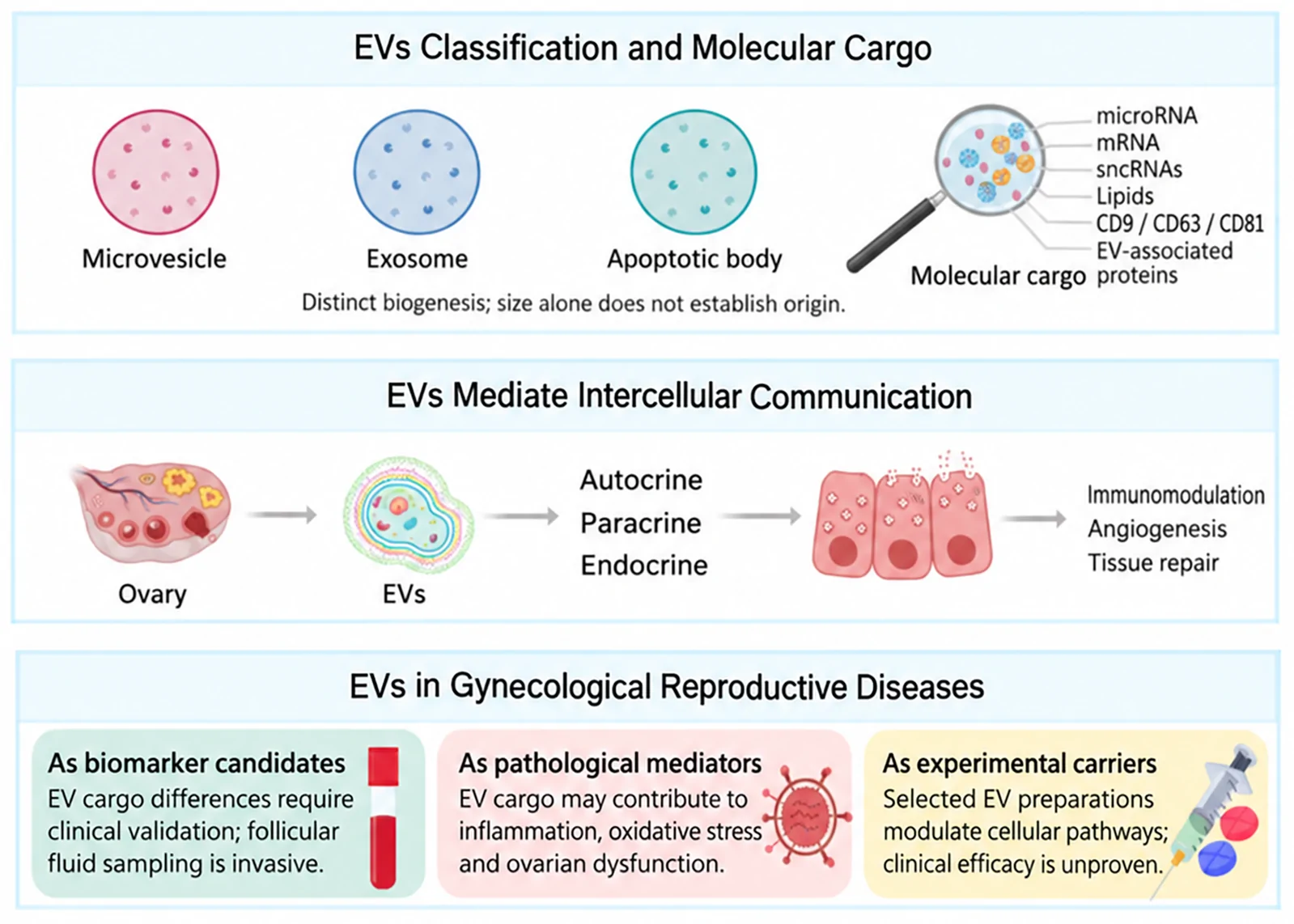
Extracellular vesicle formation, molecular cargo and intercellular communication. The upper panel presents EV populations with distinct biogenesis and examples of molecular cargo. These populations are not depicted as interconverting. CD9, CD63 and CD81 are examples of EV-associated proteins rather than universal or exosome-specific markers. The middle panel illustrates autocrine, paracrine and endocrine communication. The lower panel summarizes EVs as biomarker candidates, pathological mediators and experimental carriers. EV nomenclature follows MISEV2023 (2).

EV-associated RNA can alter recipient-cell signaling, providing a rationale for studying these particles as biomarkers and delivery systems (3). Related reproductive studies illustrate that effects can extend beyond the ovary: adipose tissue EV-associated miR-515-5p has been implicated in placental metabolism in gestational diabetes mellitus (4). Such evidence provides context but does not demonstrate efficacy in PMOS. The present review focuses on ovarian signaling, PMOS-specific experiments and the requirements for translation.

### 1.1 Review approach and scope

This article is a narrative review rather than a systematic review or meta-analysis. It organizes published clinical observational, animal and cell-culture evidence by biological question and intervention type. Mechanistic statements are linked to the relevant primary study; ovarian injury and premature ovarian insufficiency studies are identified separately as contextual evidence. No claim of exhaustive retrieval or a prospectively registered search protocol is made. Treatment studies are summarized in **Table 1**, and a descriptive MISEV2023-based appraisal of five studies with accessible main full texts is presented in Section 4.1.

**Table 1:** Experimental interventions involving extracellular vesicles in PMOS.

Study	EV source	Model and administration	Cargo or mechanism	Reproductive endpoints	Metabolic endpoints
Zhao et al. (5)	Adipose MSC EVs	Cumulus cells and letrozole-induced mice	miR-323-3p/PDCD4	Proliferation and apoptosis; ovarian/endocrine phenotype	—
Zhou et al. (6)	Follicular fluid EVs	KGN cells and letrozole-induced rats	miR-18b-5p/PTEN/PI3K-Akt-mTOR	FSH, LH, testosterone ↓; estradiol ↑	—
Cao et al. (7)	Adipose MSC EVs	PCOS rats	miR-21-5p/hepatic BTG2/IRS1-AKT	Ovarian phenotype; fertility	Metabolic disturbance/hepatic signaling
Park et al. (8)	Human MSC EVs	Androgen-producing cells and letrozole-induced mice; intravenous or intraovarian administration	Endocrine and anti-inflammatory effects	Androgen-related and reproductive endpoints	Metabolic improvement reported
Abd-Elwahab et al. (9)	MSC EVs versus conditioned medium	Rats; oral letrozole 1 mg/kg/day for 5 weeks	Comparative secretome intervention	Biochemical and ovarian histological outcomes	—
Fang et al. (10)	Brown adipose tissue EVs	C57BL/6J mice; oral letrozole 1 mg/kg/day for 21 days; EVs via tail vein, 200 µg	STAT3 ↓/GPX4 ↑	Cycles, hormones, oocyte quality, pregnancy/litter size	Body weight; insulin resistance
Mansoori et al. (11)	Menstrual blood MSC EVs	GCs from letrozole-treated rats (1 mg/kg/day, 21 days); EV exposure *in vitro*, 8 µg/mL for 24 h	Mitochondrial biogenesis/antioxidant response	Estradiol in culture; GC viability	Cellular mitochondrial endpoints
Teng et al. (12)	Bone marrow MSC EVs	NMRI mice; subcutaneous DHEA 6 mg/100 g/day for 21 days; intravenous EVs, 100 µg/kg on day 21 (as reported)	Apoptosis/vascular-marker response	Histology; corpora lutea; TUNEL	—
Teng et al. (13)	Bone marrow MSC EVs	PCOS mice	CD31/inflammation/oxidative stress	Ovarian vascular-marker outcomes	Local stress/inflammation markers
Zhou et al. (14)	Umbilical cord MSC EVs	DHEA-treated KGN cells and PCOS mice	METTL3 ↓/PINK1 m6A ↓/mitophagy ↓	GC apoptosis; ovarian function/hormones	Mitochondrial measurements
Ding et al. (15)	CP05-740Y-P umbilical cord MSC sEVs	C57BL/6 mice; DHEA 6 mg/100 g/day for 21 days; intravenous sEVs, 1×10^10^ particles/mouse; peptide loading input, 100 µg	CP05-CD63/740Y-P/PI3K-Akt	Estrous cycles; follicles; litter size	Glucose and insulin tolerance
Khorram et al. (16)	Adipose MSC exosome-like nanovesicles	NMRI mice; single subcutaneous estradiol valerate induction; intraperitoneal nanovesicles, 25–100 µg/kg, twice over 2 weeks	FOXO3/Map1lc3b/SF-1 associations	LH/FSH and estradiol; histology; injury at 100 µg/kg	—
Yuan et al. (17)	Follicular fluid EV miR-424-5p	Human follicular fluid, LPS-treated GCs and DHEA-induced rats	FGF2/FGFR1	Inflammatory/ovulation-related outcomes	—
Solati et al. (18)	Wharton’s jelly MSC EVs versus conditioned medium	Mouse COCs; oral letrozole 90 µg/kg/day for 7 days; *in vitro* EVs 5 µg/mL or CM 50 µg/mL for 24 h	IVM outcomes; maturation-gene restoration mainly reported in CM groups	IVM, viability and morphology; no *in vivo* fertility rescue	—
Chen et al. (19) [indirect]	Endogenous adipose EVs after formula treatment	Rats; letrozole + high-fat/high-sucrose diet + PEPD inhibitor for 35 days; formula treatment for 28 days	miR-27a-3p/PPARG association	Sex steroids; ovarian histology	Glucose/lipids; hepatic lipid accumulation
Wu et al. (20) [indirect]	Endogenous follicular EVs after acupuncture	Rats; intraperitoneal DHEA 60 mg/kg/day for 21 days; acupuncture for 4 weeks; KGN experiments	miR-873-5p/HMOX1/ferroptosis	Oocyte maturation; histology; GC ferroptosis	—

Model details, administration routes, doses and endpoints are included where verified from accessible primary-study records. Omitted details and “—” indicate information that could not be verified or is not applicable; they do not imply that the original study failed to report the item. No comparison of equivalent dosing or efficacy is inferred. Protein-based and particle-based doses are not interchangeable. The 100 µg peptide value for Ding et al. is loading input, not a measured delivered dose. The EV dose for Teng et al. (12) is reproduced as reported. The final two rows concern indirect modulation of endogenous EVs rather than administration of an EV product. MSC, mesenchymal stromal cell; EV, extracellular vesicle; sEV, small EV; GC, granulosa cell; COC, cumulus–oocyte complex; CM, conditioned medium; IVM, *in vitro* maturation; BAT, brown adipose tissue; DHEA, dehydroepiandrosterone; LPS, lipopolysaccharide; PEPD, prolidase; FSH, follicle-stimulating hormone; LH, luteinizing hormone. ↑, increase; ↓, decrease.

## 2 EV signaling and ovarian function

Ovarian function depends on coordinated signaling among granulosa cells, cumulus cells and oocytes. The following sections distinguish mechanistic evidence from ovarian injury models, disease-associated follicular signaling and the limitations of ovarian targeting.

### 2.1 Granulosa cell protection in ovarian injury models

EV preparations have been investigated for their ability to modify apoptosis and oxidative injury in granulosa cells. Xiao et al. (21) studied amniotic fluid stem cell-derived EVs containing miR-10a in chemotherapy-related ovarian injury. This experiment was not a PMOS model. Its relevance is that EV-associated miRNAs can contribute to follicle protection under a defined injury condition.

Zhu et al. (22) investigated human umbilical cord stromal cell-derived EV-associated circBRCA1. FTO-dependent regulation stabilized circBRCA1, which acted through the miR-642a-5p/FOXO1 axis, increasing FOXO1 and mitigating oxidative injury. This work concerned oxidative stress and premature ovarian insufficiency-related biology, not direct proof of PMOS treatment efficacy. Likewise, hsa_circ_0002021 was studied in premature ovarian failure-related granulosa cell dysfunction (23). These adjacent disease models provide mechanistic hypotheses that require independent testing in PMOS. **Figure 2** separates these contextual mechanisms from the PMOS-specific METTL3 pathway discussed in Section 3.2.1.

**Figure 2:**
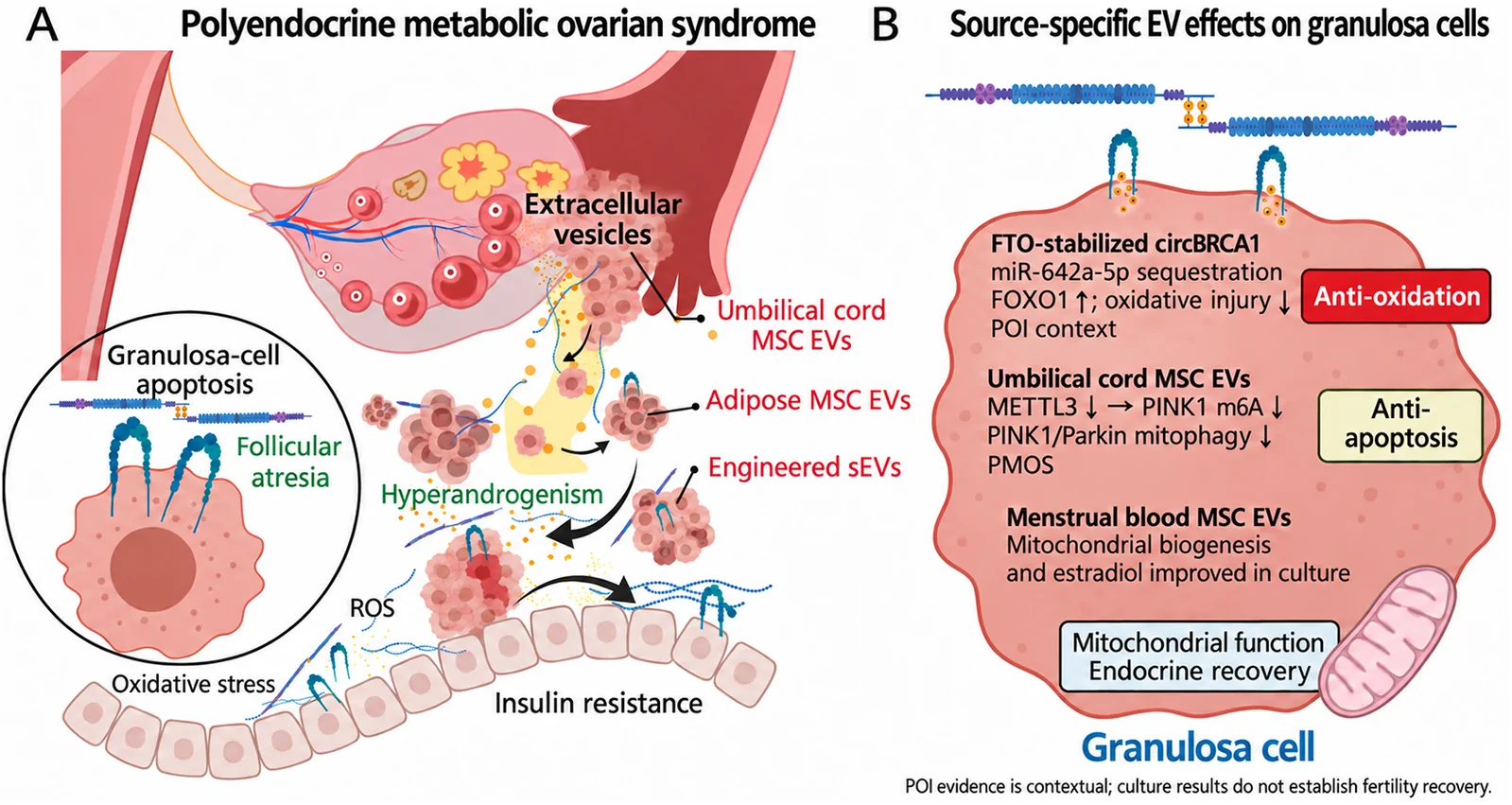
Source-specific effects of extracellular vesicles on granulosa cell function. **(A)** Schematic representation of ovarian and cellular abnormalities associated with PMOS, including granulosa cell apoptosis, follicular atresia, oxidative stress, hyperandrogenism and insulin resistance. The depicted EV preparations represent investigated intervention sources. **(B)** Independent mechanisms include FTO-stabilized circBRCA1/miR-642a-5p/FOXO1 signaling in POI-related oxidative injury research (22), inhibition of METTL3-associated PINK1 m6A modification and excessive mitophagy in PMOS experimental models (14), and mitochondrial and estradiol-related improvements after menstrual blood stromal cell EV exposure in granulosa cell culture (11). These findings arise from different experimental contexts and do not constitute a single pathway. ↑ increase; ↓ decrease. MSC, mesenchymal stromal cell; POI, premature ovarian insufficiency.

### 2.2 Communication within the follicular microenvironment

Follicular fluid EVs may influence granulosa cells, cumulus cells and oocytes, but biological activity depends on the donor state and cargo. A change in cell proliferation is not necessarily a therapeutic improvement: excessive or poorly coordinated proliferation can coexist with arrested follicular development. The same endpoint must therefore be interpreted alongside steroidogenesis, oocyte competence and follicular maturation.

Disease-associated cargo can be detrimental. Yu et al. (24) linked elevated follicular fluid EV-associated LIPE-AS1 to altered steroid metabolism and impaired oocyte maturation through a LIPE-AS1/miR-4306/LHCGR regulatory axis. Adipocyte-derived EV-associated miR-26b was also reported to promote cumulus cell apoptosis by targeting JAG1 (25). These findings show that EVs can transmit pathological signals as well as support cell survival. Detailed PMOS studies of miR-143-5p, miR-18b-5p and oocyte maturation are presented in Sections 3.1, 3.2.1 and 3.4, respectively.

### 2.3 Tissue exposure and the limits of ovarian targeting

Ovarian uptake after a particular administration regimen does not establish a general natural ovarian tropism. Experimental biodistribution studies show substantial hepatic and splenic accumulation, with distribution affected by cell source, dose, route and targeting strategy (26). Ovarian exposure should therefore be quantified relative to other organs and compared with unmodified particles and appropriate labeling controls. Local administration or improved ovarian delivery of a loaded drug cannot be generalized to all circulating EVs. The engineered carrier study discussed in Section 3.2.3 is a formulation-specific example.

## 3 Diagnostic and therapeutic evidence in PMOS

### 3.1 Biomarker candidates and pathogenic follicular cargo

Circulating and follicular fluid EV studies address different clinical questions. Willis et al. (27) reported increased circulating annexin V-positive platelet microparticles in women with PCOS. This is observational evidence about circulating particles, not a study of stromal cell EV therapy or follicular fluid RNA sequencing.

Wyse et al. (28) compared follicular fluid particle fractions and granulosa cell small RNA profiles in women with and without PCOS, stratified by adiposity. Their findings support the importance of BMI and sample fraction when interpreting RNA signatures. The reported six upregulated and sixteen downregulated small RNAs came from the overall follicular fluid comparison across particle types, rather than an exclusively sEV-specific signature. Differences between extracellular fractions and granulosa cells are consistent with selective extracellular RNA distribution, but do not alone prove a particular packaging mechanism.

Lian et al. (29) studied follicular fluid EV-associated miR-143-5p in an IVF/ICSI cohort and performed functional experiments. Elevated miR-143-5p suppressed RASAL2 and promoted granulosa cell proliferation. In a letrozole-induced rat model, inhibition of miR-143-5p increased RASAL2 and improved ovarian and hormonal abnormalities. Thus, the proliferative effect was interpreted as part of disease pathogenesis; supplementation with miR-143-5p should not be presented as therapeutic. The study supports a candidate biomarker and a possible inhibitory target, rather than a validated diagnostic test or evidence that all proliferation-promoting EVs are beneficial.

Clinical validation requires independent cohorts, prespecified thresholds and demonstration of added value beyond established clinical assessment. Adiposity, androgen phenotype, ovarian stimulation, medication exposure and infertility-related selection can affect measured cargo. Follicular fluid is generally collected during oocyte retrieval and is an invasive specimen; circulating EVs are more suitable for exploring non-invasive sampling, but disease-specific diagnostic performance remains to be established. Associations with oocyte or embryo outcomes, including LIPE-AS1, should not be equated with validated prediction of pregnancy or live birth (24).

### 3.2 Experimental EV interventions

#### 3.2.1 Local ovarian and cellular mechanisms

Zhou et al. (14) studied umbilical cord stromal cell-derived EVs in DHEA-treated KGN cells and a mouse PCOS model. EV treatment reduced METTL3 expression, PINK1 m6A modification and PINK1 transcript stability, suppressing excessive PINK1/Parkin-associated mitophagy and apoptosis. METTL3 overexpression partly reversed the EV effect. The findings support inhibition of the METTL3-associated modification pathway. Mitochondrial measurements support involvement of quality-control signaling.

Adipose stromal cell EV-associated miR-323-3p promoted cumulus cell proliferation and reduced apoptosis through PDCD4 in the study by Zhao et al. (5). This is a distinct mechanism from amniotic fluid stem cell miR-10a and from METTL3/PINK1 signaling.

Zhou et al. (6) investigated follicular fluid EV-associated miR-18b-5p in KGN cells and letrozole-induced PCOS rats. The proposed pathway involved PTEN suppression and activation of PI3K/Akt/mTOR. Treatment was associated with reduced FSH, LH and testosterone and increased estradiol. This represents one study spanning cellular and animal experiments. Yuan et al. (17) described a separate miR-424-5p/FGF2/FGFR1 mechanism relevant to ovarian inflammation. Neither finding justifies treating unselected patient follicular fluid EVs as uniformly protective.

Mansoori et al. (11) exposed granulosa cells isolated from letrozole-treated rats to menstrual blood stromal cell-derived EVs. Mitochondrial biogenesis, oxidative stress and estradiol-related endpoints improved in culture. This was not evidence of fertility rescue after EV administration to intact animals. Teng et al. (12) reported improved ovarian morphology and reduced apoptosis after bone marrow stromal cell EV administration in DHEA-treated mice. A subsequent report described reduced CD31 expression and inflammatory and oxidative stress markers (13). CD31 changes should be interpreted as vascular-marker observations rather than proof of universal pro-angiogenic activity or ovarian homing.

Khorram et al. (16) examined adipose stromal cell-derived “exosome-like nanovesicles” in an estradiol valerate-induced mouse model. Reported changes included a lower LH/FSH ratio, higher estradiol and increased FOXO3, Map1lc3b and SF-1 expression. The best histological response occurred at 75 µg/kg, whereas 100 µg/kg was associated with inflammation and partial tissue degeneration. This dose-dependent adverse finding argues against assuming that greater EV exposure is always more effective or safe. Transcript changes indicate pathway associations and do not by themselves establish cargo-specific causality.

#### 3.2.2 Metabolic mechanisms and endocrine outcomes

PMOS is not only a reproductive endocrine disorder but is often accompanied by systemic metabolic abnormalities including insulin resistance, glucose and lipid metabolism disorders, and obesity. The therapeutic effects of some EVs do not act directly on the ovary but rather indirectly improve systemic metabolic status through regulation of metabolic organs such as the liver, subsequently reversing ovarian pathological changes.

Cao et al. (7) reported that adipose stromal cell-derived EVs transferred miR-21-5p to rat liver, reduced BTG2 and activated IRS1/AKT signaling. Metabolic improvement accompanied changes in ovarian phenotype and fertility. The study supports an extraovarian mechanism and should not be used as evidence of selective ovarian delivery.

Fang et al. (10) administered brown adipose tissue-derived EVs to letrozole-induced PCOS mice. They reported improvements in insulin resistance, estrous cyclicity and fertility, lower LH and testosterone, reduced ovarian STAT3 and increased GPX4. This mechanism is separate from the miR-21-5p/BTG2/IRS1/AKT pathway.

Park et al. (8) investigated stromal cell-derived EVs in androgen-producing cells and letrozole-induced mice, using intravenous and intraovarian administration. The study provides experimental support for endocrine and reproductive effects of stromal cell EV treatment. Comparisons of conditioned medium and EV preparations also highlight the possibility that soluble secreted factors contribute to biological activity (9). Similar endpoints across studies do not establish equivalent efficacy because preparations, models and administration differ (**Table 1**).

#### 3.2.3 Engineered delivery

Ding et al. (15) used a CD63-binding anchor peptide, CP05, to attach the PI3K agonist 740Y-P to human umbilical cord stromal cell sEVs. In DHEA-induced mice, intravenous treatment improved reproductive and glucose-insulin endpoints relative to the model condition. The study reported improved ovarian delivery of 740Y-P; it also reported no difference in ovarian internalization of unmodified and engineered sEVs at the assessed time point. These observations support enhanced delivery of the peptide by the carrier, not a general increase in EV ovarian homing. Quantitative organ-level biodistribution, off-target signaling and long-term safety remain necessary for translation.

#### 3.2.4 Modulation of endogenous EV signaling

Endogenous EV modulation differs from administering an isolated therapeutic product. Chen et al. (19) reported that Bushen Huatan formula treatment in a non-obese rat model was associated with lower adipose EV miR-27a-3p and higher ovarian PPARG and insulin-signaling components, alongside endocrine and metabolic improvements. These findings support a proposed signaling mechanism rather than clinical validation of an EV therapy.

Wu et al. (20) reported that acupuncture increased follicular fluid EV-associated miR-873-5p, reduced HMOX1 and alleviated granulosa cell ferroptosis in experimental systems. Inhibition experiments supported involvement of the miRNA. This mechanism should be evaluated alongside the intervention protocol, EV characterization and alternative systemic effects. Neither study establishes therapeutic efficacy in women with PMOS. **Figure 3** summarizes the distinct intervention routes and the contrasting effects of disease-associated follicular cargo.

**Figure 3:**
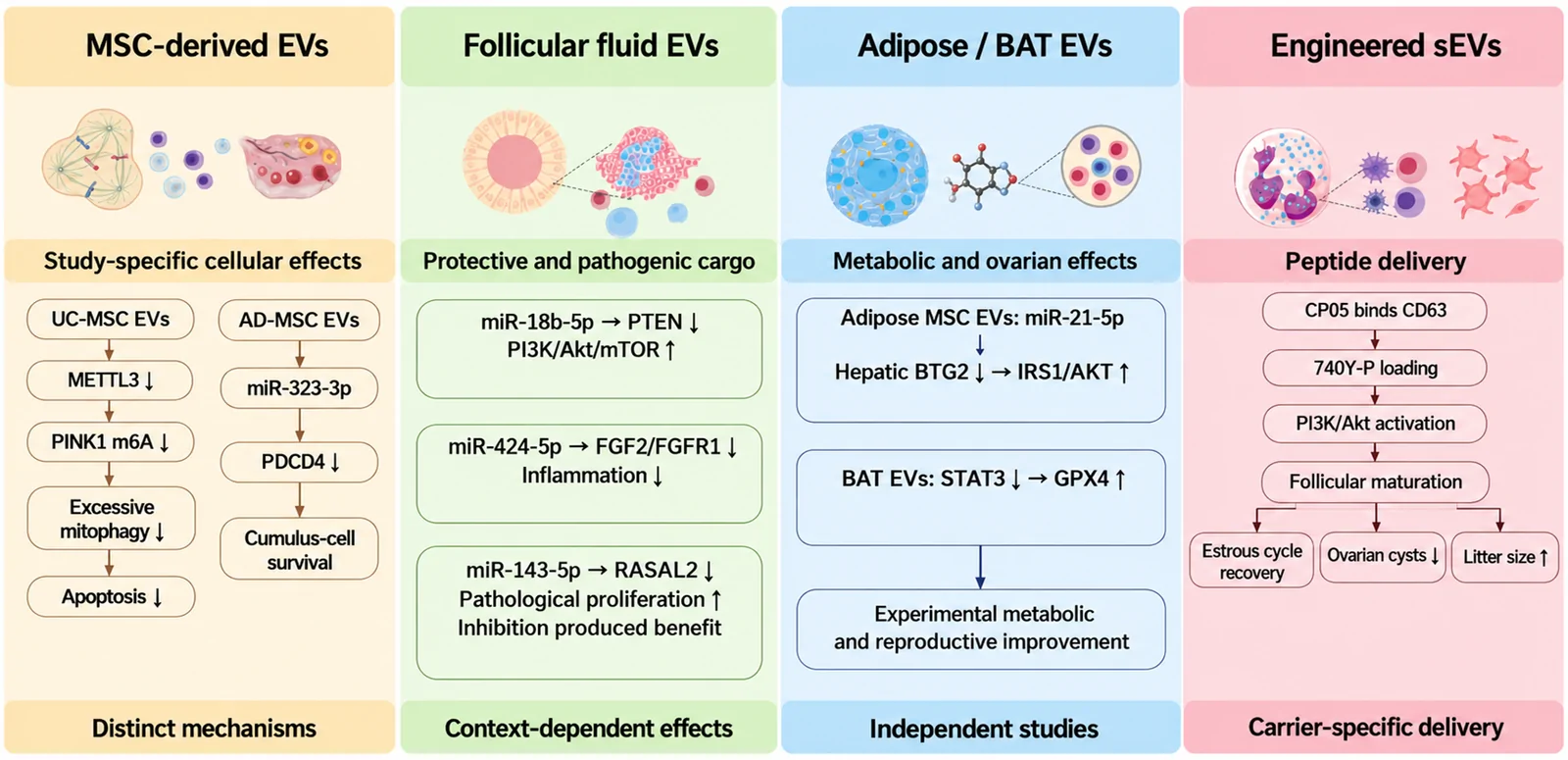
Extracellular vesicle-associated mechanisms and experimental interventions in PMOS. The four columns summarize selected MSC-derived EV mechanisms, follicular fluid EV cargo, adipose/brown adipose tissue EV effects and engineered sEV delivery. METTL3/PINK1-associated signaling (14) is distinguished from miR-323-3p/PDCD4 signaling (5). Follicular mechanisms include miR-18b-5p/PTEN/PI3K-Akt-mTOR (6), miR-424-5p/FGF2-FGFR1 (17) and pathogenic miR-143-5p/RASAL2 signaling, for which inhibition produced benefit (29). Hepatic miR-21-5p/BTG2/IRS1-AKT signaling (7) and STAT3/GPX4 signaling (10) represent independent studies. Engineered delivery uses CP05 binding to CD63 to anchor 740Y-P (15); improved peptide delivery does not establish a general increase in EV ovarian homing. ↑ increase; ↓ decrease. BAT, brown adipose tissue; MSC, mesenchymal stromal cell; sEV, small extracellular vesicle.

### 3.3 EV signaling in androgen-associated inflammation

Salehi et al. (30) linked androgen-exposed granulosa cell-associated miR-379-5p signaling to macrophage polarization and a pro-inflammatory follicular environment. The reported effect depended on the follicular context, illustrating that hyperandrogenism can alter intercellular communication rather than acting only through direct steroid toxicity. Endocrine changes observed after EV interventions are summarized in Section 3.2 and **Table 1**. Future experiments should distinguish direct effects on androgen synthesis from secondary changes following improved insulin sensitivity, inflammation or follicular development.

### 3.4 Oocyte maturation and infertility

Liu et al. (31) investigated follicular EVs from nonhyperandrogenic PCOS patients requiring IVF. Exposure of mouse oocytes to these preparations impaired maturation and was associated with increased oxidative stress and mitochondrial and spindle abnormalities. The selected clinical phenotype and cross-species assay limit generalization to all PMOS patients, but the findings demonstrate that patient-derived follicular EVs may transmit harmful signals. Together with LIPE-AS1 and miR-143-5p, these observations explain why EV source and donor phenotype matter when considering follicular fluid-based interventions.

Solati et al. (18) compared Wharton’s jelly stromal cell-derived conditioned medium and EV supplementation during *in vitro* maturation of mouse oocytes. Both CM and EV supplementation improved selected maturation-related outcomes, although CM generally showed greater benefit. Significant restoration of maturation-related gene expression was reported primarily in CM-treated groups; these findings should not be interpreted as proof of an EV-specific CDK1-dependent mechanism.

Endometrial work is adjacent evidence rather than PMOS-specific treatment evidence. Zhang et al. (32) reported improved pregnancy rates after intrauterine administration of umbilical cord stromal cell EV gel in thin-endometrium rats. It does not establish correction of endometrial receptivity in PMOS. A therapeutic strategy for PMOS-associated infertility must assess both oocyte competence and implantation outcomes in the relevant disease context.

## 4 Evidence quality and translational priorities

### 4.1 MISEV2023 based reporting appraisal

MISEV2023 recommends transparent source and separation reporting, complementary physical and molecular characterization, evaluation of relevant co-isolates, and controls that connect an observed function to EVs (2). CD9, CD63 and CD81 are examples of EV-associated membrane proteins, not universal or exosome-specific identifiers. Morphology and size alone are insufficient, and particle concentration should be distinguished from total preparation protein.

We applied these principles in a descriptive appraisal of five studies with accessible main full texts. The main-text characterization described by Teng et al. (12) includes morphology and size but does not report an EV-associated protein panel. Solati et al. (18) similarly describes TEM and DLS without an EV protein panel in the inspected main text. Mansoori et al. (11) and Wu et al. (20) report EV-associated proteins but do not describe a contaminant/negative-marker panel in the inspected main-text characterization. Ding et al. (15) reports both positive and cellular negative markers, although further cytosolic EV-marker and co-isolate assessments would strengthen interpretation. These are reporting limitations, not proof that vesicles were absent, and supplementary or inaccessible information may alter individual judgments.

For the remaining studies, incomplete access prevents a comparable full-text appraisal; they are not classified as compliant or non-compliant. Across the selected reports, major unresolved questions include endosomal provenance and exclusion of non-vesicular contributions to biological activity. Suitable controls include matched EV-depleted fractions, medium processed without producer cells, cargo loss-of-function and rescue, and labeling controls for uptake experiments. Comparing whole conditioned medium with an EV preparation does not itself isolate the contribution of EVs. A descriptive appraisal therefore improves interpretation without assigning unsupported numerical quality scores.

### 4.2 Comparability, efficacy and safety

The treatment studies use different induction methods, including DHEA, letrozole and estradiol valerate, and span cultured cells, *in vitro* oocyte maturation and intact animals. Their endpoints range from expression changes to litter size. Reproductive recovery cannot be inferred from cell viability alone, and improved glucose tolerance does not establish restoration of ovulation. **Table 1** distinguishes these outcomes and summarizes model and administration details where they could be verified from accessible records. Protein-based and particle-based doses should not be treated as interchangeable, particularly when contaminating proteins may affect the measured dose.

Source-dependent biodistribution and hepatic or splenic uptake make targeting and systemic effects central translational issues (26). Future studies should compare ovarian exposure with liver, spleen and other organs, report retention and clearance over time, and include free-label controls. For engineered products, loading efficiency, unbound drug removal, batch consistency and carrier-versus-cargo effects require separate measurement. Immunogenicity and biological stability depend on preparation and administration; they cannot be assumed to be favorable for every EV product.

Safety evaluation should include repeated dosing, inflammation, endocrine perturbation, ovarian reserve, ectopic proliferative effects and offspring outcomes. The high-dose tissue injury reported by Khorram et al. (16) illustrates why efficacy and safety must be assessed together. Human sampling studies identify associations, while experimental interventions remain preclinical; the cited evidence does not establish clinical efficacy or an approved EV treatment for PMOS.

## 5 Conclusions

EV research in PMOS connects ovarian cell signaling with systemic metabolism and offers candidate biomarkers, inhibitory targets and experimental delivery systems. The strongest interpretation is source- and context-specific: some preparations improve defined experimental endpoints, whereas disease-associated follicular cargo can impair oocyte competence or contribute to abnormal proliferation. Clinical translation requires independently validated biomarkers, reproducible EV characterization, comparable administration and dose reporting, quantitative biodistribution and long-term reproductive safety. These requirements follow directly from the limitations of the reviewed studies and should guide the next stage of research.
